# Influence of amyloid and diagnostic syndrome on non-traditional memory scores in early-onset Alzheimer’s disease

**DOI:** 10.1002/alz.13434

**Published:** 2023-08-31

**Authors:** Justin Bushnell, Dustin B. Hammers, Paul Aisen, Jeffrey L. Dage, Ani Eloyan, Tatiana Foroud, Lea T. Grinberg, Leonardo Iaccarino, Clifford R. Jack, Kala Kirby, Joel Kramer, Robert Koeppe, Walter A. Kukull, Renaud La Joie, Nidhi S. Mundada, Melissa E. Murray, Kelly Nudelman, Malia Rumbaugh, David N. Soleimani-Meigooni, Arthur Toga, Alexandra Touroutoglou, Prashanthi Vemuri, Alireza Atri, Gregory S. Day, Ranjan Duara, Neill R. Graff-Radford, Lawrence S. Honig, David T. Jones, Joseph Masdeu, Mario Mendez, Erik Musiek, Chiadi U. Onyike, Meghan Riddle, Emily Rogalski, Steven Salloway, Sharon Sha, Raymond S. Turner, Thomas S. Wingo, David A. Wolk, Maria C. Carrillo, Bradford C. Dickerson, Gil D. Rabinovici, Liana G. Apostolova, David G. Clark, the LEADS Consortium

**Affiliations:** 1 Department of Neurology, Indiana University School of Medicine, Indianapolis, Indiana, USA; 2 Alzheimer’s Therapeutic Research Institute, University of Southern California, San Diego, California, USA; 3 Department of Biostatistics, Center for Statistical Sciences, Brown University, Providence, Rhode Island, USA; 4 Department of Medical and Molecular Genetics, Indiana University School of Medicine, Indianapolis, Indiana, USA; 5 Department of Pathology, University of California – San Francisco, San Francisco, California, USA; 6 Department of Neurology, University of California – San Francisco, San Francisco, California, USA; 7 Department of Radiology, Mayo Clinic, Rochester, Minnesota, USA; 8 Department of Radiology, University of Michigan, Ann Arbor, Michigan, USA; 9 Department of Epidemiology, University of Washington, Seattle, Washington, USA; 10 Department of Neuroscience, Mayo Clinic, Jacksonville, Florida, USA; 11 Laboratory of Neuro Imaging, USC Stevens Neuroimaging and Informatics Institute, Keck School of Medicine of USC, Los Angeles, California, USA; 12 Department of Neurology, Massachusetts General Hospital and Harvard Medical School, Boston, Massachusetts, USA; 13 Banner Sun Health Research Institute, Sun City, Arizona, USA; 14 Department of Neurology, Mayo Clinic, Jacksonville, Florida, USA; 15 Wien Center for Alzheimer’s Disease and Memory Disorders, Mount Sinai Medical Center, Miami, Florida, USA; 16 Taub Institute and Department of Neurology, Columbia University Irving Medical Center, New York, New York, USA; 17 Department of Neurology, Mayo Clinic, Rochester, Minnesota, USA; 18 Nantz National Alzheimer Center, Houston Methodist and Weill Cornell Medicine, Houston, Texas, USA; 19 Department of Neurology, David Geffen School of Medicine at UCLA, Los Angeles, California, USA; 20 Department of Neurology, Washington University in St. Louis, St. Louis, Missouri, USA; 21 Department of Psychiatry and Behavioral Sciences, Johns Hopkins University School of Medicine, Baltimore, Maryland, USA; 22 Department of Neurology, Alpert Medical School, Brown University, Providence, Rhode Island, USA; 23 Department of Psychiatry and Behavioral Sciences, Mesulam Center for Cognitive Neurology and Alzheimer’s Disease, Feinberg School of Medicine, Northwestern University, Chicago, Illinois, USA; 24 Department of Neurology & Neurological Sciences, Stanford University, Palo Alto, California, USA; 25 Department of Neurology, Georgetown University, Washington D.C., USA; 26 Department of Neurology and Human Genetics, Emory University School of Medicine, Atlanta, Georgia, USA; 27 Department of Neurology, Perelman School of Medicine, University of Pennsylvania, Philadelphia, Pennsylvania, USA; 28 Medical & Scientific Relations Division, Alzheimer’s Association, Chicago, Illinois, USA

**Keywords:** Alzheimer’s, amnestic, amyloid, memory, neuropsychology, PCA, PPA, primacy, recency

## Abstract

**INTRODUCTION:**

The Rey Auditory Verbal Learning Test (RAVLT) is a useful neuropsychological test for describing episodic memory impairment in dementia. However, there is limited research on its utility in early-onset Alzheimer’s disease (EOAD). We assess the influence of amyloid and diagnostic syndrome on several memory scores in EOAD.

**METHODS:**

We transcribed RAVLT recordings from 303 subjects in the Longitudinal Early-Onset Alzheimer’s Disease Study. Subjects were grouped by amyloid status and syndrome. Primacy, recency, J-curve, duration, stopping time, and speed score were calculated and entered into linear mixed effects models as dependent variables.

**RESULTS:**

Compared with amyloid negative subjects, positive subjects exhibited effects on raw score, primacy, recency, and stopping time. Inter-syndromic differences were noted with raw score, primacy, recency, J-curve, and stopping time.

**DISCUSSION:**

RAVLT measures are sensitive to the effects of amyloid and syndrome in EOAD. Future work is needed to quantify the predictive value of these scores.

## 1 | INTRODUCTION

Neuropsychological tests have traditionally served as the foundation for the detection and monitoring of Alzheimer’s disease (AD) and related disorders (ADRD).^1,2^ Depending on the type of neuropsychological test, digital analysis can provide additional scores based on speech quality,^3,4^ acceleration of motion,^5^ or timing measurements (e.g., during writing tasks^6^).

Early detection of ADRD will depend on understanding relationships between biological markers of disease and cognitive test scores, whether novel, digital, or traditional. The study of preclinical, dominantly inherited AD shows that Mini-Mental State Exam (MMSE) and story recall scores for mutation carriers diverge from scores of non-carriers about 8 years before estimated onset.^7^ The presence of apolipoprotein *ε*4 (*APOE4*) alleles is associated with changes in verbal fluency, including the timings and lexical frequencies of words generated.^8,9^ In late-onset AD (LOAD), amyloid deposition is associated with deficits in story memory delayed free recall^10^ and composite visual-verbal memory scores.^11^ While the current work focuses on audio recordings of a memory test, we are interested in automatic extraction of diagnostically valuable scores, including traditional scores that do not depend on sophisticated technology.

LOAD and early-onset AD (EOAD) is characterized clinically by comparable impairment of verbal episodic memory,^12–17^ although patients with LOAD may exhibit greater semantic impairment and those with EOAD may exhibit greater deficits of visuoconstructive and executive function,^18^ or praxis.^19^ Atypical presentations, including logopenic progressive aphasia,^20^ posterior cortical atrophy,^21^ and a dysexecutive subtype^22^ are more common in EOAD than in LOAD, although the amnestic presentation is still the most common.^23^

Novel methods for scoring episodic memory tests could establish stronger relationships between cognition and biomarkers. Serial position effects (SPEs) aim to capture the tendencies of remembering (or forgetting) certain parts of a word list. Primacy and recency are the tendencies to remember words from the beginning and end of lists, respectively, with recency typically showing the highest rate of recall, although recency effects may be attenuated by aging, cognitive impairment, or imposition of a delay.^24–29^ Studies consistently show primacy to be diminished in subjects with mild cognitive impairment (MCI) or LOAD,^30,31^ and to be associated with the integrity of the hippocampus.^32^ Some recent research links SPEs to AD biomarker status. Among individuals with amnestic MCI, primacy scores are lower in those positive for beta-amyloid.^33^ A ratio of recency scores from a story recall task (immediate recency/long-delay recency) is a strong predictor of total tau, phosphorylated tau, and neurofilament light in the cerebrospinal fluid (CSF).^34^ To our knowledge, SPEs have not been specifically evaluated in EOAD, and one goal of the current work is to evaluate their utility in this population.

Owing to the importance of word-list learning tasks for AD diagnosis, we seek to evaluate traditional and novel measures of word list recall in a cross-sectional sample of individuals with early-onset dementia. We focus specifically on the influence of amyloid positivity on scores across the eight tasks of the Rey Auditory Verbal Learning Test (RAVLT).^35,36^ We hypothesize that presence of amyloid will be associated with reduction in a speed score analogous to one from our previous work (detailed in Section 2.4).^37^ Because the RAVLT test is not administered within a fixed time frame (unlike verbal fluency), we calculated two additional scores based on timing. One of these scores is the duration of each task. The other score is a novel ratio indexing an individual’s perception of time, which may be altered by hippocampal changes in LOAD.^38–40^ We hypothesize these timing-based scores will reflect slowing in subjects with amyloid. Regarding SPEs, we expect a reduction in primacy and J-curve (refer to Section 2.4). We hypothesize a lower primacy score and higher J-curve score in amnestic subjects. In addition, we explore the patterns of performance seen among the various early-onset dementia presentations, which are variably associated with AD and non-AD pathology.

## 2 | METHODS

### 2.1 | Subjects

At the time of this analysis, the Longitudinal Early-Onset Alzheimer’s Disease Study (LEADS)^41^ had enrolled 91 participants with normal cognition and 314 participants with cognitive impairment. Age below 65 was a criterion for entry into the study. We selected 303 subjects with either normal cognition (*N* = 89) or cognitive impairment (*N* = 214) who had been audio recorded while undergoing the RAVLT at the baseline evaluation. Cognitively impaired subjects were categorized according to severity, that is, dementia or MCI, using criteria from the National Institute on Aging-Alzheimer’s Association (NIA-AA).^41–43^ Cases of cognitive impairment were also assigned to diagnostic categories: amnestic, non-amnestic, posterior cortical atrophy (PCA), and logopenic primary progressive aphasia (PPA) following standardized criteria.^41,44,45^ All cognitively impaired subjects underwent amyloid positron emission tomography scans and were classified as “amyloid-positive” or “amyloid-negative” according to the U.S. Food and Drug Administration-approved criteria for visual interpretation.^41,46^ For the biomarker analysis, we combined cognitively normal (all amyloid-negative) and amyloid-negative, cognitively impaired (EOnonAD) subjects. Although we lack biomarker-based diagnoses for the EononAD subjects, we consider these subjects critical for discerning the influence of amyloid on our outcomes of interest.

### 2.2 | RAVLT

The RAVLT consists of eight tasks with auditory stimulus presentation and verbal responses from the participant: five consecutive learning trials of a 15-word list “A,” a 15-word list “B” interference trial, a short-delay free recall of list A immediately succeeding the interference trial, and a long-delay free recall of list A (approximately a 30-min delay) after the short-delay free recall. Cued recognition is performed in written format and was not recorded or included in this analysis. We obtained a total of 2315 RAVLT task recordings (average of 7.64 per subject). Some task recordings were missing because the participant elected not to finish the test. Note that all eight tasks have the same range of potential scores, 0 to 15 words. For each outcome measure, all 2315 tasks were entered into a single statistical model.

This study was conducted in accordance with the Declaration of Helsinki and was approved by the Institutional Review Board (IRB) at Indiana University as the central IRB for the study. All subjects or their legally authorized representatives provided written informed consent.

### 2.3 | Data pre-processing

We obtained a preliminary transcription of each RAVLT audio file using the Amazon Web Services Transcribe tool. To improve accuracy, we provided the Transcribe tool with a list of the 30 RAVLT stimulus words. We manually corrected the timings and contents of these automatic transcriptions using a custom MATLAB (Natick, MA) audiovisual transcription tool. For the purpose of calculating two of the time-based scores, we marked the duration of each task during the transcription process. We marked the beginning of each task immediately following the reading of the word list (or clarification of instructions if needed). We marked the end of each task using the moment before the participant confirmed they were finished or the moment the test administrator began the next task. To account for potential influence from the test administrator, we also marked any prompts given during each task (e.g., “Any more words?,” “Is that all?”). Prompts were found in 808 tasks of the 2315 total tasks (34.9%).

### 2.4 | Scoring

We included four count-based scores for each task in this study:

1. Raw score is the total number of valid words produced in each task. We analyze raw score as a dependent variable and include it as a covariate in the evaluation of other scores.
2. Primacy is calculated as the total number of correct words produced from the first third (i.e., first five words) of a given word list.
3. Recency is calculated as the total number of correct words produced from the final third (i.e., last five words) of a given word list.
4. J-curve is calculated by subtracting recency from primacy, and ranges from −5 to +5. It is so named because of the J shape of the SPE histogram.

We included three timing-based scores, as follows:

1. Duration is defined as the total time in seconds that a subject takes to recall a given word list.
2. We explore here a novel timing-based score, the “stopping time,” that likely relates to a participant’s perception of time. Stopping time ($t_{s}$) is calculated as $t_{s}=\left(t-t_{n}\right)/t$, where t is defined as the total duration of the task in seconds and $t_{n}$ is defined as the point in time of the last valid word’s offset. This score can be viewed as a percentage of the total task time that a participant requires to decide there are no more words accessible for recall. If a participant fails to recall any words, the stopping time is equal to 1. Division by zero is not an issue as the total duration for a task is never zero seconds.
3. A speed score was calculated as in previous work on verbal fluency.^47,48^ Briefly, the entire set of inter-word intervals (i.e., durations between valid words) is subjected to a sequence of three transformations: first, by taking the 4th root to mitigate positive skew, second, normalizing the values to lie between 0 and 1, and third, subtracting from 1. Thus, the very fastest transition receives a full point, while the slowest transition receives zero points. An individual’s speed score is the sum of these transformed durations. The speed score is thus a modified raw score where a higher number represents more words with faster response times.

**FIGURE 1:**
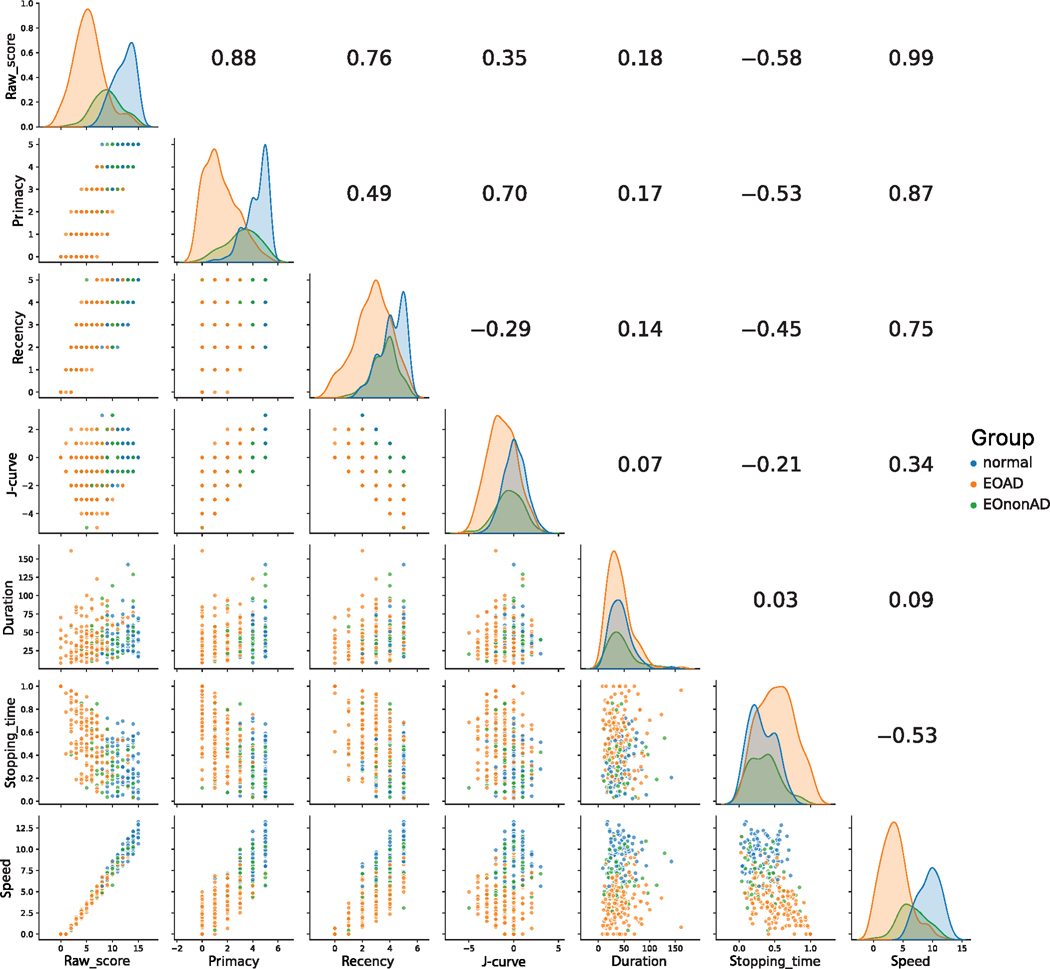
Scatterplot of trial 5. Data matrix for the trial 5 task, with data points in the categories of cognitively normal, EOAD, and EOnonAD. The upper triangle depicts Pearson’s *r* between corresponding scores. The lower triangle depicts scatter plots between corresponding scores. KDEs are on the diagonal. Abbreviations: EOAD, early-onset Alzheimer’s disease; EOnonAD, cognitively impaired; KDE, Kernel density estimate.

**FIGURE 2:**
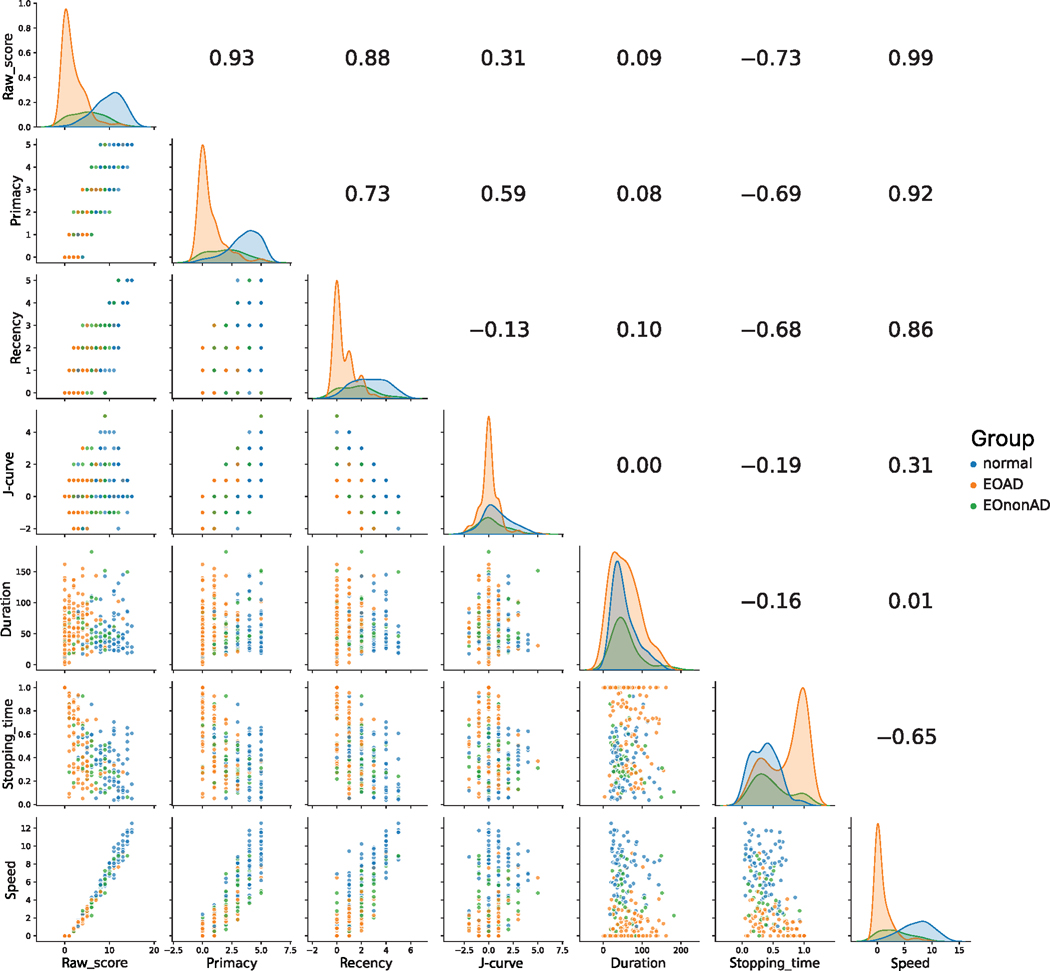
Scatterplot of long delay. Data matrix for the long-delay task, with data points in the categories of cognitively normal, EOAD, and EOnonAD. The upper triangle depicts Pearson’s *r* between corresponding scores. The lower triangle depicts scatter plots between corresponding scores. KDEs are on the diagonal. KDEs are on the diagonal. Abbreviations: EOAD, early-onset Alzheimer’s disease; EOnonAD, cognitively impaired; KDE, Kernel density estimate.

Although all RAVLT tasks were incorporated into each statistical model, we selected two of the RAVLT tasks for further illustration (list A, learning trial 5, and list A, delayed recall). The outcome variable values for these two tasks were tabulated (separated by amyloid status and clinical syndrome) and illustrated in Figures 1 and 2. The figures were formatted as matrices, with scatter plots between two scores on the lower triangular portion, correlation coefficients on the upper triangular portion, and Kernel density estimates (KDEs) along the diagonal.

### 2.5 | Statistical analyses

We fit a linear mixed-effects model for each of the seven scores, entering data on all participants and all eight RAVLT tasks into each model. Clinical syndrome (control, amnestic, PCA, PPA, non-amnestic), presence of dementia, and amyloid status (based on cohort assignment: EOAD and EOnonAD) were entered as predictor variables. We added age, education, and sex as nuisance covariates. For scores that were sensitive to timing (duration, stopping time, and speed), we included the number of prompts from the test administrator. Knowing that our non-traditional scores would be correlated with raw score, we wished to account for these correlations to the extent permitted by our statistical methodology. For example, raw score was included as a covariate for all three timing-based outcomes. The same approach could not be used for SPE scores, however, in order to ensure that raw score did not account for all the variance in SPE models. Instead, for primacy, we covaried only for the score on the final ten items of the list. Similarly, for recency we covaried with the score from the first ten words, and for J-curve, we covaried with the score from the middle five words. This way, we could covary for general learning capacity without controlling for performance on the exact items incorporated into the SPE score. Random intercepts for participant and task were included in all models. Incorporation of these random effects permits fitting a single model that simultaneously accounts for random variation in participants and RAVLT tasks while providing more traditional regression results (“fixed” effects) that quantify average relationships between each predictor and the dependent variable. Each model uses the normal group as the reference. However, all models were repeated using three of the four syndromes as a reference to examine every possible contrast. In total, there were 28 models (7 scores × 4 syndrome categories). Because of the small PCA and PPA sample sizes, we ran a supplemental analysis in which the PCA and PPA groups were combined with the non-amnestic group.

## 3 | RESULTS

### 3.1 | Subjects

Table 1 shows demographic and biomarker data on the participants, broken down by clinical syndrome. Most participants (168, 78.5%) had an amnestic presentation. Twenty-four (11.2%) were non-amnestic, 10 (4.7%) presented with PCA, and 12 (5.6%) presented with PPA. Our sample included 89 (29.4%) cognitively normal controls (CN). CN and participants with PCA exhibited a female predominance, while those with PPA and non-amnestic presentation exhibited a slight male predominance. When grouped by cohort assignment, EOAD is roughly equal (48.8% male) and EOnonAD exhibits a male predominance (65.4%). Most individuals in each of the four syndromic categories were positive for amyloid, but the proportions were highest in the amnestic and PCA groups. Dementia was more common than MCI among the cognitively impaired subjects as a whole and within each syndrome. We performed one-way analysis of variance to assess for group differences in terms of age and education and Chi-squared or Fisher’s exact test to assess for group differences in terms of severity.

### 3.2 | Scores

Figure 1 illustrates data from learning trial 5 of list A across the entire sample. The strongest correlation is between the raw and speed scores (*r* = 0.99). These scores correlated strongly with primacy and recency (*r*-values ranging from 0.75 to 0.88). Primacy and J-curve scores were strongly correlated (*r* = 0.70), as one would expect, since J-curve is calculated by subtracting recency from primacy. KDEs revealed stronger separation of CN controls from EOAD individuals than what is seen between controls and EOnonAD individuals, with the exceptions of duration, stopping time, and J-curve. For duration, there was no appreciable separation among groups. For both stopping time and J-curve, there was appreciable separation of EOAD from the other two groups.

Figure 2 illustrates data from long-delay free recall of list A. The overall pattern is similar to what was observed in Figure 1, but the separation between EOAD and the control group was starker. The main exceptions are duration and J-curve, for which there was no appreciable separation among groups.

Table 2 shows the means and standard deviations of the scores on the tasks of learning trial 5 and long delay free recall, broken down by biomarker status and clinical syndrome. In general, amyloid-positive (EOAD) individuals performed worse on each measure.

### 3.3 | Linear mixed-effects models

Table 3 shows the betas and standard errors for the linear mixed effects models. The RAVLT scores are used as the dependent variable with amyloid status, syndrome, and nuisance variables used as covariates.

Nuisance covariates (age, education, sex) did not exhibit a strong influence on the scores. However, age and sex were significantly associated with raw score. A higher age was associated with a 0.05 reduction in raw score (*β* = −0.05 [0.02], *t*(293) = −2.08, 95% CI [−0.09, −0.003], *p* = 0.0381). Those with female sex scored higher by 0.55 points across tasks (β = 0.55 [0.23], t(293) = 2.41, 95% CI [0.11, 0.99], *p* = 0.0167).

#### 3.3.1 Raw score

Presence of amyloid was associated with a 2.11 point reduction in raw score (*β* = −2.11 [0.33], *t*(294) = −6.39, 95% CI [−2.75, −1.47], *p* < 0.0001). Dementia status was associated with a reduction in raw score (*β* = −1.64 [0.30], *t*(294) = −5.56, 95% CI [−2.21, −1.07], *p* < 0.0001). When contrasted with controls, all syndromes except for PCA were associated a reduction in raw score (coefficients ranging from −1.61 to −2.38). The smallest reduction was seen in non-amnestics and the largest in PPA (non-amnestics: *β* = −1.61 [0.52], *t*(290) = −3.10, 95% CI [−2.62, −0.61], *p* = 0.0021; PPA: *β* = −2.38 [0.64], *t*(299) = −3.71, 95% CI [−3.62, −1.14], *p* = 0.0002). Changing the reference to contrast syndromes with one another showed that subjects with PCA had a greater raw score than amnestic subjects (*β* = 1.24 [0.63], *t*(290) = 1.98, 95% CI [0.03, 2.45], *p* = 0.049).

#### 3.3.2 | Primacy

Amyloid was associated with a reduction in primacy score (*β* =−0.76 [0.13], *t*(283) =−6.06, 95% CI [−1.01, −0.52], *p* < 0.0001). The raw score covariate (see Section 2.5) was associated with an increment in primacy score of 0.14 (*β* = 0.14 [0.01], *t*(2255) = 10.67, 95% CI [0.12, 0.17], *p* < 0.0001). Dementia was associated with a reduction in primacy (*β* =−0.50 [0.11], *t*(282) =−4.41, 95% CI [−0.71, −0.28], *p* < 0.0001). All clinical syndromes except for PCA were associated with reductions in primacy score, with the largest reduction seen in amnestic (*β* =−0.82 [0.14], *t*(280) =−5.92, 95% CI [−1.09, −0.55], *p* < 0.0001). Changing the reference to contrast syndromes with one another showed that PCA and non-amnestic presentations showed greater primacy scores compared with the amnestic presentation (*β*= 0.47 [0.24], *t*(273) = 1.99, 95% CI [0.01, 0.93], *p* = 0.0471) and (*β* = 0.41 [0.16], *t*(271) = 2.55, 95% CI [0.10, 0.72], *p* = 0.0113), respectively.

#### 3.3.3 | Recency

Amyloid was associated with a reduction in recency score of (*β* = −0.47 [0.12], *t*(284) = −3.98, 95% CI [−0.70, −0.24], *p* = 0.0001). The “raw score” covariate (see Section 2.5) was associated with an increment in recency score (*β* = 0.09 [0.01], *t*(2034) = 6.35, 95% CI [0.06, 0.11], *p* < 0.0001). Dementia was associated with a reduction in recency (*β* = −0.43 [0.11], *t*(280) = −4.06, 95% CI [−0.63, −0.22], *p* = 0.0001). PPA and non-amnestic presentation were associated with a significant reduction in recency score, with PPA associated with the largest effect (*β* = −0.61 [0.23], *t*(285) = −2.68, 95% CI [−1.05, −0.17], *p* = 0.0077). When amnestic presentation was used as the reference, PPA was associated with a significant reduction in recency score (*β* = −0.45 [0.21], *t*(289) = −2.20, 95% CI [−0.85, −0.06], *p* = 0.0283). When PCA was used as the reference, PPA was associated with a significant reduction in recency score (*β* = −0.63 [0.29], *t*(279) = −2.15, 95% CI [−1.20, −0.06], *p* = 0.0320).

#### 3.3.4 J-curve

Amyloid status was not a significant predictor of J-curve score. The raw score covariate (see section 2.5) was positively associated with J-curve score (*β* = 0.15 [0.03], *t*(2110) = 5.12, 95% CI [0.09, 0.21], *p* < 0.0001). Of the syndromes, only amnestic presentation was associated with a reduction in J-curve score (*β* = −0.53 [0.16], *t*(297) = −3.22, 95% CI [−0.84, −0.21], *p* = 0.0014). Non-amnestic presentation was associated with higher J-curve score than amnestic presentation in the pairwise comparisons (*β* = 0.54 [0.19], *t*(283) = 2.88, 95% CI [0.18, 0.90], *p* = 0.0043).

#### 3.3.5 | Duration

Each prompt from the test administrator increased duration of tasks by 12.12s (seconds) on average (*β* = 12.12 [0.85], *t*(2214) = 14.29, 95% CI [10.48, 13.82], *p* < 0.0001). Each item correctly retrieved increased duration by 1.92s (*β* = 1.92 [0.18], *t*(2234) = 10.55, 95% CI [1.57, 2.28], *p* < 0.0001). The various clinical groups did not differ from the CN group, nor from one another.

#### 3.3.6 | Stopping time

Amyloid increased stopping time by 5% (*β* = 0.05 [0.02], *t*(301) = 3.04, 95% CI [0.02, 0.08], *p* = 0.0025). Each prompt from the test administrator increased stopping time by 4% (*β* = 0.04 [0.01], *t*(1614) = 4.38, 95% CI [0.02, 0.06], *p* < 0.0001). Increasing raw score reduced stopping time by 5% per word retrieved (*β* = −0.05 [0.00], *t*(385) = −27.00, 95% CI [−0.05, −0.04], *p* < 0.0001). All syndromic diagnoses were associated with a reduction in stopping time. Beta coefficients ranged from −0.06 (PPA: *β* = −0.06 [0.03], *t*(304) = −2.01, 95% CI [−0.13, −0.003], *p* = 0.0448) to −0.15 (PCA: *β* = −0.15 [0.03], *t*(282) = −4.41, 95% CI [−0.22, −0.09], *p* < 0.0001). Pairwise contrasts revealed that PCA was associated with a lower stopping time than amnestic (*β* = −0.08 [0.03], *t*(282) = −2.59, 95% CI [−0.14, −0.02], *p* = 0.0100). PPA subjects presented with a higher stopping time with reference to PCA subjects (*β* = 0.09 [0.04], *t*(296) = 2.15, 95% CI [0.01, 0.17], *p* = 0.0323).

#### 3.3.7 | Speed

Each prompt diminished speed (*β* = −0.07 [0.02], *t*(2301) = −3.65, 95% CI [−0.12, −0.03], *p* = 0.0003). Raw score was positively associated with speed score (*β* = 0.78 [0.00], *t*(994) = 185.62, 95% CI [0.78, 0.79], *p* < 0.0001). All clinical syndromes were associated with reductions in speed score, with betas ranging from −0.28 for amnestic presentation (*β* = −0.28 [0.07], *t*(300) = −4.06, 95% CI [−0.41, −0.14], *p* = 0.0001) to −0.41 for PPA (*β* = −0.41 [0.12], *t*(303) = −3.48, 95% CI [−0.64, −0.18], *p* = 0.0006). Changing the reference to contrast syndromes with one another did not yield any additional significant results.

#### 3.3.8 Supplementary results

See Supplementary Table e-1 for results of the analysis with PCA, PPA, and non-amnestic groups combined.

Supplementary Table e-2 shows the random intercept values for the tasks, across the seven outcome variables. These intercept values show that scores tend to improve with each learning trial and decline with delay. Of note, the random effects for primacy, recency, and J-curve replicate other research suggesting that delay has a greater impact on recency.^27^

## 4 | DISCUSSION

We examine the RAVLT performance of cognitively impaired individuals from the LEADS, most of whom have biomarker evidence of Alzheimer disease, using the traditional raw score and six non-traditional scoring methods. Of these additional methods, three consist of scoring subsets of the word lists (primacy, recency, and J-curve). The other three depend on precise measurement of the timings of responses. These measurements are greatly facilitated by the use of automatic speech recognition, technology that is critical if speech- and language-based digital biomarkers are to be applied to cognitive screening on a massive scale.

As the value of digital biomarkers depends ultimately on their relationship to pathology, one of our key objectives is to evaluate the effect of amyloid on various scores of an episodic memory test. We find that regardless of clinical presentation, RAVLT raw scores across the eight tasks are on average 2.11 points lower for amyloid-positive individuals than for amyloid-negative individuals. This finding is supported in part by Stricker et al.,^49^ where long-delay free recall is lower among amyloid-positive subjects. Clark et al.^50^ found that elevated amyloid in CSF is associated with an accelerated decline in total output of all learning trials over time compared with biomarker-negative subjects. With regard to SPEs, we found that brain amyloidosis is associated with significant reductions in both the primacy and recency subset scores, but not with the difference between the two (J-curve score).

In previous work, we found that scores based on speed of word generation were more informative than raw scores for predicting onset of cognitive impairment from semantic fluency,^48^ especially in the subset of individuals most likely to have AD. However, in the current analysis, we do not find that amyloid positivity is associated with identically derived speed scores for the RAVLT. The apparent divergence of these findings might be due to differences between the verbal fluency and list-learning tasks. The former task is thought to engage executive processes and semantic memory, while the second is supported chiefly by episodic memory. It is possible that timing differences emerge for verbal fluency, but not the RAVLT, due to the relatively large number of valid items that may potentially be retrieved during verbal fluency. Another possibility is that verbal fluency is administered with a fixed time frame (usually 60 seconds) and the participant is aware that a higher score is dependent on rate of output. The RAVLT does not have a time constraint and therefore is completed without such urgency.

The proportion of the total duration between generation of the final valid word and the end of the task (stopping time) is associated with biomarker status, as amyloid-positive individuals take longer to finish searching for additional words. Critically, this effect is present despite inclusion of raw score and number of prompts in the model. However, there is some uncertainty regarding the normal range for stopping time. If the stopping time is too short, the participant potentially misses out on recalling more words. If the stopping time is too long, the participant exhibits inefficiency. This inefficiency could be due to lack of attention, reduced awareness of the passage of time,^40^ or anosognosia (lack of awareness of the memory deficit). Further, the presence of repetitions or intrusions during the recall period could create a false sense of progress that may contribute to the relative delay to end the task.

Some investigators have proposed that primacy scores relate to the integrity of episodic memory, while recency scores relate to simple attention.^51^ Some of our findings support this view. Comparing the effects within the syndromic groups for primacy and recency, for example, we observe different patterns between the amnestic and non-amnestic groups. The effect (*β*) for primacy is strongest in the amnestic group (−0.82), while the effect for recency in this group is among the lowest (−0.16), and the raw difference between the two coefficients is of the largest magnitude (0.66). Moreover, only the amnestic group differs significantly from controls in terms of J-curve. In the non-amnestic group, the effect for primacy is among the lowest (−0.41), while the effect for recency is among the highest (−0.38, a difference of 0.03), with the non-amnestic group differing significantly from the amnestic group on primacy. A very different pattern is seen within the PPA group, for which the coefficient magnitude of primacy (−0.75) is quite large and recency (−0.61) is the largest among all syndromes. Impairment of attention has been observed in subjects with amnestic and non-amnestic MCI,^52^ and also subjects with (logopenic variant) PPA,^53^ which our recency findings support. However, the large effect of PPA syndrome on recency score could be due to the combination of attention and language deficits. Finally, the PCA group exhibits low magnitudes for both coefficients (−0.35 and −0.02).

The patterns observed across these clinical syndromes may be accounted for by variable contributions of dysfunction within mesial temporal regions supporting episodic memory,^54^ dorsolateral frontal regions supporting attention to verbal material,^55^ and posterior superior temporal or inferior parietal regions important for the phonological loop of verbal working memory.^5^ The pattern for the amnestic group suggests relatively isolated hippocampal dysfunction (leading to low primacy and relatively higher recency scores). Low primacy and recency scores among the individuals in the PPA group may result from involvement of both frontal and temporo-parietal regions supporting verbal working memory, leading to a secondary impairment of verbal episodic memory. The non-amnestic group may suffer from isolated frontal dysfunction, disrupting attention in a way that equally affects primacy and recency. Finally, the PCA group exhibits the least evidence of damage to these structures supporting verbal short- and long-term memory, and manifests with the highest primacy and recency scores.

We observed a sex effect for raw score, with women producing more words in general. This finding agrees in part with Van der Elst et al.,^57^ who report that women produced more words, but also note an effect of age (likely due to inclusion of participants with a broader range of ages). We observed no effects of age and sex with regard to primacy and recency.

The limitations of this work point to the need for further research. First, the sample sizes in some clinically defined groups (PCA and PPA) are small. Our ability to detect effects of these syndromes on the outcome measures is therefore weakened and may have led to Type II errors for some scores. Second, this analysis of performance in early-onset cognitive decline may not generalize to patients with late onset decline. Patients with early-onset dementia manifest more heterogeneously than those with late-onset dementia, even when evaluating only those with biomarker support for Alzheimer disease.^5^ Though short- and long-delay free recall scores are similar between EOAD and LOAD, learning trial scores are significantly lower in subjects with EOAD.^59^ Third, RAVLT raw scores were one of three memory measures inspected by members of the consensus team when determining the severity and nature of cognitive impairment. Thus, there is a risk of circularity where the raw scores are concerned. However, this concern does not apply to the influence of amyloid status, nor to the non-traditional outcomes of interest (e.g., SPEs). Fourth, some of the measures we report here depend on measuring the duration of each task (duration and stopping time). In most cases, the end of each task is unambiguous, as the participant states explicitly that he or she could think of no more target words. However, in some cases, the test administrator (perhaps following nonverbal cues from the participant) opts to move on to the next task. Thus, it is possible that the test administrators truncate some of the tasks, leading to measurement of a smaller stopping time. It is therefore possible that the true effect of our predictor variables (including amyloid positivity) might be larger than what we report here. This concern is somewhat mitigated by our observation that all three KDEs for duration overlap neatly (see Figures 1 and 2); thus, there is little evidence that test administrators inadvertently shorten the duration of tasks for EOAD individuals in a systematic way. If the stopping time is to be explored further as a digital marker of amyloid status, it will be helpful to modify the test administration to ensure a uniform protocol for ending each task. Finally, while we demonstrate several statistically significant effects based only on the presence of amyloid, it is likely that concomitant tau aggregation is present in most of these individuals and may bear a stronger relationship to cognitive performance.

In summary, we identify four episodic memory scores with a statistically significant relationship to brain amyloid: the raw score, primacy and recency scores, and stopping time. Patterns of performance with primacy and recency may reflect the extent of pathologic involvement of cerebral regions supporting episodic memory, language, or attention. Future work with detailed brain imaging will help to confirm or falsify these hypotheses. Stopping time is a novel score based on timing measurements that should be investigated for its ability to predict biomarker status of patients with suspected neurodegenerative disease.

## Supplementary Material

Table 2

Table 1

Supp

## Figures and Tables

**TABLE 1:** LEADS participants.

	All	Normal	Amnestic	PCA	PPA	Non-amnestic
***N***	303	89	168	10	12	24
**Age**	58.44 (5.14)	57.17(5.98)	58.98 (4.54)	60.93 (2.49)	59.77 (3.69)	57.69(5.97)
**Education (years)**	15.74 (2.37)	16.54(2.24)	15.42 (2.34)	15.00 (2.05)	15.92 (2.22)	15.21 (2.41)
**Sex (M:F)**	144:159	31:58	86:82	2:8	8:4	17:7
**Severity (MCI:Dem)**	78:136	–	66:102	2:8	5:7	5:19
**Amyloid (N:P)**	52:162	–	39:129	1:9	4:8	8:16

*Notes:* Continuous variables are displayed as mean (standard deviation).

Abbreviations: Dem, dementia; F, female; M, male; LEADS, Longitudinal Early-Onset Alzheimer’s Disease Study; MCI, mild cognitive impairment; N, negative; P, positive, PCA, posterior cortical atrophy; PPA, primary progressive aphasia.

**TABLE 2:** Scores for RAVLT trial 5 and long-delay recall, stratified by presence or absence of amyloid.

	Trial 5, amyloid negative				
	Normal (*N* = 86)	Amnestic (*N* = 38)	PCA(*N* = 1)	PPA(*N* = 4)	Non-amnestic (*N* = 8)
**Raw_score**	12.20 (2.03)	9.39 (2.55)	7.00 (0.00)	7.75 (4.38)	8.38 (1.93)
**Primacy**	4.30 (0.89)	3.26 (1.31)	3.00 (0.00)	2.75 (1.48)	2.88 (1.05)
**Recency**	4.17 (0.88)	3.76 (0.84)	3.00 (0.00)	2.75 (1.30)	3.50 (0.50)
**J-curve**	0.13 (1.19)	−0.50 (1.59)	0.00 (0.00)	0.00 (0.71)	−0.62 (1.22)
**Duration**	42.93 (20.54)	43.42 (22.91)	23.10 (0.00)	66.19 (38.34)	35.91 (19.76)
**Stopping_time**	0.32 (0.18)	0.33 (0.21)	0.39 (0.00)	0.55 (0.09)	0.34 (0.16)
**Speed**	9.48 (1.85)	6.92 (2.20)	5.19 (0.00)	5.30 (3.45)	6.08 (1.84)
	Trial 5, amyloid positive				
	Normal	Amnestic (*N* = 124)	PCA(*N* = 9)	PPA(*N* = 6)	Non-amnestic (*N* = 16)
**Raw_score**	–	5.31 (2.81)	7.56(2.31)	5.17(4.78)	5.81 (3.28)
**Primacy**	–	1.36(1.21)	2.44(1.17)	1.67(1.70)	1.88 (1.45)
**Recency**	–	2.83 (1.30)	3.44 (0.96)	2.00(1.53)	2.25 (1.15)
**J-curve**	–	−1.47(1.53)	−1.00(1.25)	−0.33(1.37)	−0.38 (1.45)
**Duration**	–	41.25 (30.86)	50.33 (30.94)	41.63 (24.12)	44.91 (19.64)
**Stopping_time**	–	0.53(0.25)	0.38 (0.23)	0.51 (0.29)	0.52 (0.20)
**Speed**	–	3.56 (2.17)	5.06(1.98)	3.25 (3.61)	3.88 (2.69)
	Long delay, amyloid negative				
	Normal (*N* = 83)	Amnestic (*N* = 35)	PCA(*N* = 1)	PPA(*N* = 4)	Non-amnestic (*N* = 7)
**Raw_score**	9.81 (3.22)	5.49 (3.45)	2.00 (0.00)	5.00 (5.52)	4.57 (3.16)
**Primacy**	3.57(1.31)	2.03 (1.38)	2.00 (0.00)	1.75 (2.05)	2.00 (1.85)
**Recency**	2.70 (1.36)	1.74 (1.34)	0.00 (0.00)	1.75 (2.05)	1.29 (0.70)
**J-curve**	0.87 (1.39)	0.29(1.47)	2.00 (0.00)	0.00 (0.00)	0.71 (1.58)
**Duration**	54.22 (30.65)	56.10 (36.51)	19.73 (0.00)	85.21 (37.08)	49.94 (21.94)
**Stopping_time**	0.37 (0.21)	0.48 (0.28)	0.53 (0.00)	0.60 (0.34)	0.38 (0.29)
**Speed**	7.18 (2.82)	3.46 (2.61)	0.71 (0.00)	2.91 (3.63)	2.74 (2.37)
	Long delay, amyloid positive				
	Normal	Amnestic (*N* = 114)	PCA(*N* = 9)	PPA(*N* = 6)	Non-amnestic (*N* = 16)
**Raw_score**	–	1.57(2.19)	3.89 (3.75)	3.17(4.60)	3.50 (3.00)
**Primacy**	–	0.51 (0.94)	1.33(1.33)	1.00 (1.83)	1.31(1.26)
**Recency**	–	0.50 (0.81)	1.00 (0.94)	0.83 (1.46)	0.94 (0.83)
**J-curve**	–	0.01 (0.96)	0.33 (0.67)	0.17(0.69)	0.38 (0.93)
**Duration**	–	59.73 (48.42)	57.56(36.14)	45.12 (22.41)	63.81 (36.27)
**Stopping_time**	–	0.77 (0.29)	0.54 (0.37)	0.53 (0.35)	0.54 (0.30)
**Speed**	–	0.70(1.27)	2.34 (2.68)	1.85 (3.35)	1.82 (1.93)

*Note*: Data are presented asmean (standard deviation).

Abbreviations: PCA, posterior cortical atrophy; PPA, primary progressive aphasia; RAVLT, Rey Auditory Verbal Learning Test.

**TABLE 3:** Syndrome regression coefficients (standard error) for each RAVLT score.

	Raw_score	Primacy	Recency	J-curve	Duration	Stopping_time	Speed
**Age**	−0.05 (0.02)*	−0.01 (0.01)	−0.01 (0.01)	−0.003 (0.01)	−0.04 (0.24)	−0.002 (0.001)	−0.003 (0.004)
**Amyloid (positive)**	−2.11 (0.33)*	−0.76 (0.13)*	−0.47 (0.12)*	−0.24 (0.15)	5.82 (3.61)	0.05 (0.02)*	−0.02 (0.06)
**Amnestic**	−2.24 (0.36)*	−0.82 (0.14)*	−0.16(0.13)	−0.53(0.16)*	6.88 (3.98)	−0.07 (0.02)*	−0.28 (0.07)*
**PCA**	−1.00 (0.71)	−0.35 (0.27)	0.02 (0.25)	−0.28 (0.31)	7.06 (7.72)	−0.15 (0.03)*	−0.40 (0.13)*
**PPA**	−2.38 (0.64)*	−0.75 (0.24)*	−0.61 (0.23)*	−0.10 (0.29)	10.36 (6.97)	−0.06 (0.03)*	−0.41 (0.12)*
**Non-amnestic**	−1.61(0.52)*	−0.41 (0.20)*	−0.38 (0.18)*	0.01 (0.23)	7.91 (5.66)	−0.10 (0.03)*	−0.36 (0.10)*
**Dementia (status)**	−1.64 (0.30)*	−0.50 (0.11)*	−0.43 (0.11)*	−0.05 (0.13)	−3.38 (3.22)	0.01 (0.01)	0.01 (0.05)
**Number of prompts**	−	−	−	−	12.12 (0.85)*	0.04 (0.01)*	−0.07 (0.02)*
**Education**	0.05 (0.05)	0.02 (0.02)	0.02 (0.02)	0.01 (0.02)	−0.84 (0.52)	−0.003 (0.002)	0.01 (0.01)
**Gender (female)**	0.55 (0.23)*	0.02 (0.09)	0.13 (0.08)	−0.12 (0.10)	2.26(2.47)	−0.01 (0.01)	−0.01 (0.04)
**Raw score**	−	0.14 (0.01)*	0.09 (0.01)*	0.15 (0.03)*	1.92 (0.18)*	−0.05 (0.002)*	0.78 (0.004)*
**R_m_^2^, R_c_^2^**	0.43,0.80	0.46,0.70	0.21,0.57	0.09,0.39	0.10,0.70	0.47,0.52	0.98,0.99

*Note*: Shown are the estimates (standard errors) of the linear mixed effects models. The raw score covariate for primacy, recency, and J-curve only take into account the portion of rawscore not accounted for by these scores (e.g., the rawscore covariate for primacy includes the score on the last 10 items of the list).

Abbreviations: PCA, posterior cortical atrophy; PPA, primary progressive aphasia; RAVLT, Rey Auditory Verbal Learning Test.

* *p* < 0.05.

## Highlights

- RAVLT patterns characterize various presentations of EOAD and EOnonAD
- Amyloid impacts raw score, primacy, recency, and stopping time
- Timing-based scores add value over traditional count-based scores

## RESEARCHINCONTEXT

1. **Systematic review**: Literature review was done mainly through PubMed, Google Scholar, and bibliographies of relevant papers. Though the RAVLT is heavily cited, there are few relevant articles pertaining to atypical AD presentations. Inferences were made from general characteristics of these syndromes where necessary.
2. **Interpretation**: The work presented here demonstrates the sensitivity of RAVLT scores, based on timing and serial position, for showing differences among amyloid positive or negative individuals. Further, some scores reliably differ among subjects with varying presentations of early-onset Alzheimer’s disease.
3. **Future direction**: The application of these scores to subjects with late-onset Alzheimer’s disease needs to be studied in order to determine whether these findings are generalizable. Further, the field would benefit from quantifying the predictive value of such scores. Finally, building on previous literature, identification of asymptomatic amyloid-positive subjects using serial or novel time-based scores needs to be assessed.
