# Supplementary material for: Influence of amyloid and diagnostic syndrome on non-traditional memory scores in early-onset Alzheimer’s disease
Source: Alzheimers Dement. Author manuscript; Available in PMC 2024 Nov 1. (PMC10855009; doi:10.1002/alz.13434)
Supplement: Table 2 [file NIHMS1950138-supplement-Table_2.docx]

| **Table e-2. Random intercept values, sorted highest to lowest by task** | | | | | | | | | | | | | | | | | | | | | | | |
| --- | --- | --- | --- | --- | --- | --- | --- | --- | --- | --- | --- | --- | --- | --- | --- | --- | --- | --- | --- | --- | --- | --- | --- |
| **Raw Score** | |  | **Primacy** | |  | **Recency** | |  | **Jcurve** | |  | **Duration** | |  | **Stopping Time** | | |  | | **Speed** | | |  |
| T5 | 2.282 |  | T5 | 0.508 |  | T5 | 0.722 |  | SD | 1.015 |  | LD | 16.913 |  | T5 | 0.012 |  | | T4 | | 0.078 |  |  |
| T4 | 1.907 |  | T4 | 0.458 |  | T4 | 0.635 |  | LD | 0.925 |  | SD | 6.509 |  | T2 | 0.011 |  | | T5 | | 0.072 |  |  |
| T3 | 1.116 |  | T3 | 0.314 |  | T3 | 0.478 |  | T3 | -0.138 |  | T5 | -0.902 |  | T4 | 0.009 |  | | T2 | | 0.034 |  |  |
| T2 | 0.022 |  | SD | 0.223 |  | T2 | 0.273 |  | T4 | -0.176 |  | T4 | -2.148 |  | T1 | 0.006 |  | | T3 | | 0.032 |  |  |
| SD | -0.496 |  | T2 | 0.023 |  | LB | 0.140 |  | T2 | -0.189 |  | T3 | -2.606 |  | LB | 0.003 |  | | T1 | | -0.004 |  |  |
| LD | -1.028 |  | LD | -0.023 |  | T1 | -0.150 |  | T5 | -0.221 |  | LB | -3.568 |  | LD | -0.001 |  | | LB | | -0.053 |  |  |
| T1 | -1.858 |  | T1 | -0.546 |  | SD | -0.973 |  | T1 | -0.292 |  | T2 | -5.696 |  | T3 | -0.004 |  | | LD | | -0.062 |  |  |
| LB | -1.946 |  | LB | -0.957 |  | LD | -1.126 |  | LB | -0.924 |  | T1 | -8.502 |  | SD | -0.036 |  | | SD | | -0.096 |  |  |
| Abbreviations: T1-T5, learning trials 1-5; LB, list B; SD, short delay; LD, long delay.  Note that the recency effect is particularly impacted by delay. | | | | | | | | | | | | | | | | | | | | | | | |
