# Supplementary material for: Influence of amyloid and diagnostic syndrome on non-traditional memory scores in early-onset Alzheimer’s disease
Source: Alzheimers Dement. Author manuscript; Available in PMC 2024 Nov 1. (PMC10855009; doi:10.1002/alz.13434)
Supplement: Table 1 [file NIHMS1950138-supplement-Table_1.docx]

| **Table e-1. Amnestic vs. Non-amnestic regression coefficients (standard error) for each RAVLT score** | | | | | | | |
| --- | --- | --- | --- | --- | --- | --- | --- |
|  | **raw_score** | **primacy** | **recency** | **jcurve** | **duration** | **stopping_time** | **speed** |
| **Age** | -0.05(0.02)* | -0.01(0.01) | -0.01(0.01) | -0.003(0.01) | -0.04(0.24) | -0.002(0.001) | -0.003(0.004) |
| **Amyloid (Positive)** | -2.09(0.33)* | -0.76(0.13)* | -0.45(0.12)* | -0.25(0.15) | 5.78(3.59) | 0.05(0.02)* | -0.02(0.06) |
| **Amnestic** | -2.27(0.36)* | -0.83(0.14)* | -0.18(0.13) | -0.52(0.16)* | 6.96(3.97) | -0.07(0.02)* | -0.28(0.07)* |
| **Non amnestic** | -1.71(0.44)* | -0.49(0.17)* | -0.37(0.16)* | -0.08(0.20) | 8.44(4.80) | -0.10(0.02)* | -0.38(0.08)* |
| **Dementia** | -1.62(0.29)* | -0.48(0.11)* | -0.42(0.11)* | -0.04(0.13) | -3.47(3.20) | 0.01(0.01) | 0.01(0.05) |
| **Number of prompts** | - | - | - | - | 12.13(0.85)* | 0.04(0.01)* | -0.07(0.02)* |
| **Education** | 0.05(0.05) | 0.02(0.02) | 0.02(0.02) | 0.01(0.02) | -0.83(0.52) | -0.003(0.002) | 0.01(0.01) |
| **Gender (Female)** | 0.59(0.22)* | 0.03(0.08) | 0.15(0.08) | -0.13(0.10) | 2.19(2.43) | -0.01(0.01) | -0.01(0.04) |
| **Raw Score** | - | 0.14(0.01)* | 0.09(0.01)* | 0.15(0.03)* | 1.92(0.18)* | -0.05(0.002)* | 0.78(0.004)* |
| **R_m_^2^, R_c_^2^** | 0.42, 0.80 | 0.46, 0.70 | 0.21, 0.57 | 0.09, 0.39 | 0.10, 0.70 | 0.46, 0.52 | 0.98, 0.99 |
| Shown are the Estimates (Standard Errors) of the linear mixed effects models. Both groups, PCA (Posterior Cortical Atrophy) and PPA (Primary Progressive Aphasia), are combined with the non-amnestic group. The "raw score" covariate for primacy, recency, and jcurve only take into account the portion of raw score not accounted for by these scores. | | | | | | | |
